# Interactive virtual assistance for mental health promotion and self-care management in elderly with type 2 diabetes (IVAM-ED): study protocol and statistical analysis plan for a randomized controlled trial

**DOI:** 10.1186/s13063-024-08055-3

**Published:** 2024-03-21

**Authors:** Frederico Ludwig da Costa, Lucas Strassburger Matzenbacher, Vicenzo Gheno, Maria Antônia Bertuzzo Brum, Laura Gomes Boabaid de Barros, Isabela Semmelmann Maia, Luiza Machado Blank, Lucas Friedrich Fontoura, Janine Alessi, Gabriela Heiden Telo

**Affiliations:** 1 Post-Graduate Program in Medicine and Health Sciences, Pontifícia Universidade Católica Do Rio Grande Do Sul, Porto Alegre, Brazil; 2 Internal Medicine Division, Hospital São Lucas – Pontifícia Universidade Católica do Rio Grande do Sul, Porto Alegre, Brazil; 3 School of Medicine, Pontifícia Universidade Católica Do Rio Grande Do Sul, Porto Alegre, Brazil; 4 Medicine and Health Sciences Graduate Program, Pontifícia Universidade Católica Do Rio Grande Do Sul, Porto Alegre, Brazil

**Keywords:** Alexa, Elderly, Healthy aging

## Abstract

**Background:**

With one in five individuals aged 65 or older living with type 2 diabetes worldwide, it is crucial to acknowledge and address the challenges faced by this population. In this context, our study aims to evaluate the efficacy of a behavioral intervention model delivered through a smart speaker on mental health and diabetes self-care in the elderly with diabetes.

**Methods:**

This is a single-center, pragmatic, parallel two-arm open randomized clinical trial involving elderly patients with type 2 diabetes. We plan to enroll a total of 112 individuals who will be randomized 1:1 to receive the Smart Speaker EchoDot 3rd Gen device (Amazon Echo®) for home use (intervention arm) or to maintain usual care (control arm). The primary outcome is mental distress, assessed using the 20-item Self Reporting Questionnaire (SRQ-20) after a 12-week intervention period. Secondary outcomes include quality of life, adherence to diabetes self-care behaviors, perception of stress, glycemic control, blood pressure, and lipid profile. Analysis of covariance (ANCOVA) will be used to evaluate the effects of the intervention on the outcomes.

**Discussion:**

This study assesses the effectiveness of an interactive virtual assistance system for enhancing mental health and glycemic control among elderly individuals with type 2 diabetes. The findings may introduce smart speakers as a valuable tool for promoting diabetes-related self-care in this population.

**Trial registration:**

ClinicalTrials.gov NCT05329376. Registered on 15 April 2022. Enrollment began on 20 June 2023 and the last update of protocol was on 13 December 2023.

**Supplementary Information:**

The online version contains supplementary material available at 10.1186/s13063-024-08055-3.

## Introduction

### Background and rationale

In recent centuries, the global population has experienced significant demographic shifts marked by an aging population, a consequence of rising life expectancy. In 1960, individuals aged 65 or older constituted 5% of the global population. Nowadays, this demographic group accounts for 10% of the population, and projections from the United Nations Population Prospect anticipate that by 2070, it will reach 20% of the world population [1].

As the global population ages, the prevalence of diabetes in the elderly is emerging as a critical public health concern. With one in five individuals aged 65 or older currently living with type 2 diabetes worldwide, representing almost 150 million people, it is important to recognize and address the challenges faced by this population [2, 3].

The comorbidity of diabetes in the elderly is associated with an increased risk of both microvascular and macrovascular diabetes complications, as well as a greater risk of premature death [4, 5]. Additionally, individuals with diabetes in this age group face an elevated risk of geriatric syndromes, including cognitive decline, functional disability, polypharmacy, frailty, depression, and diabetes-related distress [4, 5]. In this context, the use of strategies that can simplify healthcare, exploring innovative possibilities for diabetes management in the elderly, becomes essential.

The utilization of voice-activated technology and similar devices for assisting individuals with type 2 diabetes and the elderly has garnered widespread international interest. Despite substantial efforts to develop these technologies for healthcare enhancement, their adoption remains limited, highlighting the need for more comprehensive initiatives to fully utilize their potential. In this context, smart speakers and virtual assistant devices emerge as promising tools to enhance mental health, diabetes management, and overall quality of life in the elderly population.

These devices may play a crucial role in improving adherence to pharmacological treatment, addressing the fact that almost 50% of the elderly are non-adherent to medication regimens, often due to forgetfulness [6]. Additionally, a recently published study in JAMA revealed promising findings concerning the use of smart speakers in diabetes management, as it found that a voice-based conversational AI application was associated with improvement in time to optimal insulin dose and insulin adherence, although it included only 32 subjects [7].

Beyond medication management, virtual assistant devices may offer a solution to combat feelings of loneliness, as demonstrated by a feasibility study implementing smart speakers in older individuals, where half of the participants felt more connected to the outside world using the device [8].

Despite many possible applications, evidence on the efficacy of these technologies in the elderly population is limited. Therefore, we developed a model of behavioral intervention delivered through the Smart Speaker Echo Dot 3rd Gen (Amazon Alexa) to promote knowledge and skills necessary for mental health promotion and diabetes self-management in this population.

## Objectives

This study is designed to evaluate the effect of an interactive virtual assistance system delivered through Smart Speaker EchoDot 3rd Gen (Amazon Echo®) in (1) mental health, (2) glycemic control, (3) blood pressure, (4) lipid profile, (5) diabetes self-care behavior and (6) quality of life in elderly people with type 2 diabetes.

## Trial design

This is a single-center, pragmatic, volunteer-driver, registry-based, parallel two-arm (intervention-to-control group ratio = 1:1) open superiority randomized clinical trial.

## Methods

We followed the Standard Protocol Items: Recommendations for Interventional Trial (SPIRIT) 2013 guideline [9] when writing this protocol and the detailed SPIRIT checklist is available (Supplementary Table S1).

### Trial settings and recruitment

This study is a single-center trial, and all procedures will be conducted at the Center for Clinical Research of Hospital São Lucas (HSL), in southern Brazil, under the supervision of the Pontifícia Universidade Católica do Rio Grande do Sul (PUCRS). Participants will be recruited through two different strategies:

1. *Screening of records from the outpatient clinics of HSL:* Records of the outpatient clinics of HSL will be screened by a trained recruiter who will collect basic information about the inclusion criteria (date of birth, type 2 diabetes diagnosis, and city of residence). Individuals who meet the basic inclusion criteria will receive a phone call for an explanation about the study. Those who express interest in participating will undergo an assessment to determine eligibility.
2. *Social media advertising:* An electronic form via Google Forms® will be advertised via Instagram®. Interested volunteers who meet the basic inclusion criteria will receive a phone call to provide additional information about the study and to assess eligibility.

Subjects who do not respond to the initial phone call will be contacted through WhatsApp® message and will receive three phone call attempts at three different times, for a total of nine attempts. If there is no response after a total of nine unsuccessful contact attempts, the subject will be considered unreachable and ineligible for the study.

### Eligibility criteria

#### Inclusion

1. Being 65 years of age or older;
2. Having a diagnosis of type 2 diabetes;
3. Residing in Porto Alegre or metropolitan region;
4. Presenting availability of Wi-Fi connection at home; and
5. Presenting availability to participate in the proposed face-to-face evaluation and accept receiving one visit for installation of the device.

#### Exclusion

1. Having an interactive virtual home assistance device at the time of enrollment;
2. Having cognitive impairments or severe hearing impairments that prevent adequate interaction with outcome assessors and the application of follow-up questionnaires; or
3. Residing in regions of difficult access.

### Baseline evaluation and informed consent

Subjects who meet the eligibility criteria will be considered eligible for randomization and will have a baseline evaluation scheduled. Prior to collecting any data, the outcome assessor will obtain informed consent from participants and only those who provide it will proceed with the baseline evaluation and randomization.

### Randomization and blinding

Individuals who provide informed consent will undergo randomization through a 1:1 randomization sequence generated using Research Randomizer software (https://www.randomizer.org/). To ensure proper implementation, all aspects of the randomization process, including sequence generation and result disclosure, will be carried out by an independent researcher (JA) who will not be related to the study recruitment and outcome assessments. The randomization result will be disclosed only after the end of the baseline evaluation through a text message. Therefore, participants and outcome assessors will be blinded only in the baseline evaluation.

Additionally, researchers responsible for performing statistical analysis (LSM and FLB) will also be blinded. To ensure this, before extracting the data from the database, random codes for encoding the study groups will be generated by the same independent researcher responsible for allocation (JA). The disclosure of group assignments will only occur after all analyses have been completed.

### Interventions

#### Intervention group (Alexa’s group)

Individuals assigned to the intervention group will receive the Smart Speaker EchoDot 3rd Gen (Amazon Echo®) device (i.e., Alexa) for home use, installed during a home visit following the baseline evaluation. The device will be programmed using a standard model developed by the research team, applied to each patient. The proposed model involves automatic interactions between the device and the patient, comprising:

1. *Medication reminders*: The device will be programmed to automatically issue reminders at specified times for patients to take their medications and insulin, following the medical prescription provided by the attending physician.
2. *Glucose test reminders*: Automatic reminders for glucose testing will also be programmed, aligning with each patient’s individual testing frequency and routine.
3. *Educational health tips*: At two pre-defined times during the day, set in consultation with the patient during installation, the device will emit a sound alert and deliver daily flash briefings centered on health education messages. These briefings will include a greeting message, three messages related to health education, and a farewell message. The health education-related messages comprise a total of 42 unique messages developed by the research team, divided into two groups:
4. Twenty-eight messages that will be repeated every 4 weeks, one message each day, so that each message will be played three times over the 12-week study period. A detailed version of the phrases is available in Supplementary Table S2 (English, translated) and S3 (original version in Brazilian Portuguese).
5. Fourteen messages divided into 7 sets of 2 messages that will be repeated every week, two messages each day, so that each message will be played 12 times throughout the study. A detailed version of the phrases is available in Supplementary Table S4 (English, translated) and S5 (original version in Brazilian Portuguese).
6. *Weekly educational podcasts*: The device will be programmed to play 12 weekly educational podcast episodes, each lasting 5 min, recorded by the research team. These episodes cover four key themes: Physical Exercise, Diabetes Self-care, Mental Health, and Healthy Eating Habits, with three episodes dedicated to each theme. A detailed description of each episode is provided in Supplementary Table S6. Each weekly episode will be automatically played twice during the week, at times pre-defined by the patient during the device installation.
7. *Good morning and good night routine*: The good morning routine activates when the patient says “Alexa, good morning,” delivering the weather forecast for the patient’s region. Similarly, the good night routine is initiated by the patient saying “Alexa, good night,” playing a playlist of low-volume music.

In addition to the pre-determined programmed model, the individual will also receive a user manual for the device containing information about the main device commands (for example, “Alexa, play music”), common possible errors and their solutions, and a list of skills (for example, religious, games, culture, stories, and trivia) available for use on the device to encourage the use of additional functions based on the patient’s preferences. If there is any issue with the device, an internet error occurs, or the device remains offline, a new home visit will be scheduled to correct the problem, and the need for a new contact will be recorded.

#### Control group (usual care)

Patients assigned to the control group will be instructed to maintain their usual healthcare routine. Additionally, they will receive a booklet with general information and a QR code to access the research group’s website. On this website, they will have access to podcast episodes and the phrases that were automatically provided via Alexa device to participants in the intervention group.

#### Follow-up phone calls

Follow-up of participants will be maintained through five follow-up phone calls, conducted every 2 weeks. Additionally, participants from the control group will receive an extra phone call, equivalent to the device installation process, to ensure equivalent contact in both groups. A detailed timeline for these calls is provided in the following sections. The purpose of the follow-up phone calls is to ensure ongoing monitoring, and no behavioral or motivational interventions will be conducted during them.

### Outcomes

#### Primary and secondary outcomes

The primary outcome is mental distress. The six secondary outcomes are quality of life, adherence to diabetes self-care behavior, perception of stress, blood pressure, lipid profile, and glycemic control. All outcomes will be assessed at baseline (week − 1) and 12 weeks after the starting of intervention, at the final evaluation (week 12). A detailed description of each outcome and how it will be measured is presented in Table 1. All questionnaires and scores were previously validated to the population and language of our study [10–13].

**Table 1:** Primary and secondary outcomes of the study

Variable	Description
**Primary outcome**	
Mental distress measured by the SRQ-20 questionnaire	Depression, anxiety, and other common mental health disorder symptoms will be assessed using the total score on the Brazilian version of the Self Reporting Questionnaire 20 (SRQ-20). The outcome will be presented as a total score ranging from 0 to 20 points, with higher scores indicating greater mental distress
**Secondary outcomes**	
Quality of Life assessed using the SF-36 questionnaire	Total score on the Brazilian version of the 36-Item Short Form Health Survey (SF-36) Questionnaire. It evaluates the quality of life across eight domains: Functional Capacity, Limitation due to physical aspects, Pain, General health status, Vitality, Social aspects, Emotional aspects, and Mental health. The outcome will be presented as a total score ranging from 0 to 100, with higher scores indicating lower quality of life
Adherence to diabetes self-care behaviors assessed using the SCI-R questionnaire	Brazilian version of the Self-Care Inventory Revised (SCI-R) questionnaire, which reflects the follow-up of treatment recommendations and lifestyle habits related to diabetes care in the 2 months prior to the application. The outcome will be presented as a total score ranging from 11 to 55 points, with higher scores indicating a higher level of care
Perception of stress assessed using the PSS questionnaire	Total score on the Brazilian version of the Perceived Stress Scale (PSS) questionnaire. The outcome will be presented as a total score ranging from 0 to 56 points, with higher scores indicating a greater level of stress
Blood pressure	Systolic and diastolic blood pressure will be assessed using an appropriate sphygmomanometer according to the circumference of the arm. Three measures will be made for each subject and the mean of the three repeated measures will be considered
Glycemic control	Glycated hemoglobin (HbA1c) dosage performed using high-performance liquid chromatography will be considered for glycemic control evaluation
Lipid profile	Total cholesterol, HDL-c, LDL-c and triglycerides dosage

#### Adverse event monitoring

No adverse events are expected to occur as a result of the intervention. However, there is potential for participants to experience stress related to the intervention, which will be recorded as part of the primary outcome of the study. Furthermore, given that the study involves blood collection, there is a possibility of harm not directly linked to the intervention. Anticipated adverse effects such as hematoma and local pain will be monitored. Any serious adverse effects beyond these will be recorded and reported, although such occurrences are considered highly unlikely. Additionally, during the follow-up phone calls, participants will be asked about their well-being and if they have experienced any changes or symptoms since the start of the study. This information will be documented to ensure the ongoing safety and monitoring of the participants, even though a specific adverse event classification will not be applicable in this study.

### Study assessments and timeline

**Fig. 1:**
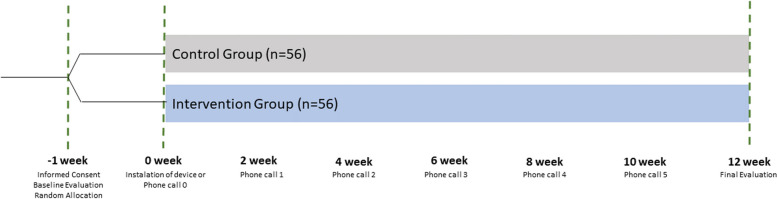
Study timeline

**Fig. 2:**
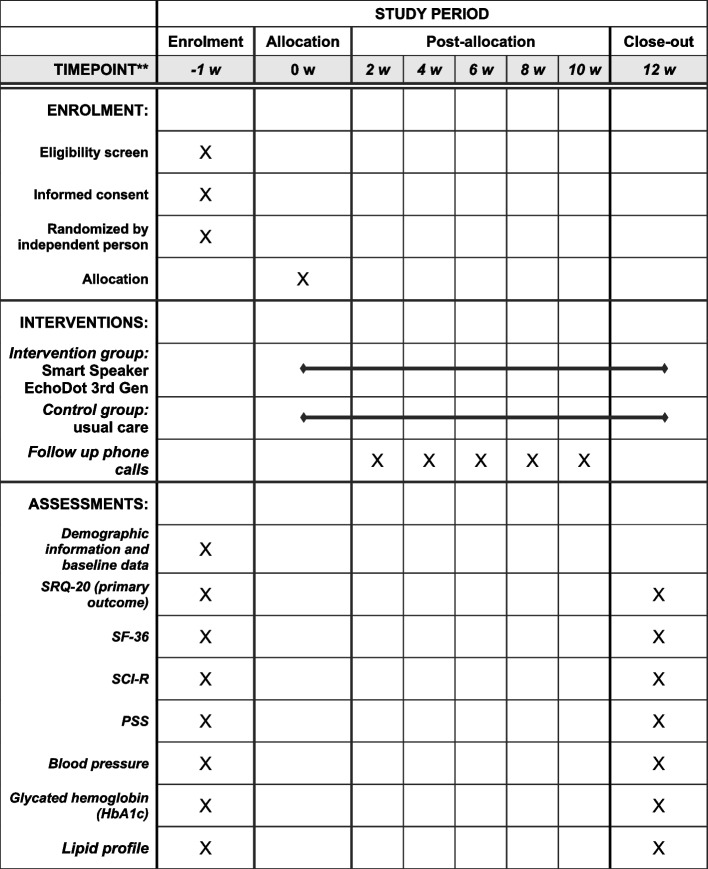
Schedule of enrolment, interventions, and assessments

All study participants will be expected to attend two in-person follow-up visits: one at baseline (week − 1) and another at the final evaluation (week 12). Additionally, participants in the intervention group will receive a home visit for the installation of the device, while participants in the control group will receive an extra follow-up phone call during the same timeframe to ensure equivalent contact (week 0). A detailed timeline is available in Fig. 1. Specific items to be evaluated during each appointment, including duration of visits and assessment time windows, as well as the logistics of the follow-up phone calls are available in Fig. 2 and at Supplementary Table S7 and S8, respectively.

### Outcome assessors

Seven members of the research team (FLC, LSM, VG, MABB, ISM, LBB, and LMB) will be responsible for conducting all study visits and follow-up phone calls, as well as screening and recruiting participants. To minimize bias, all team members received formal training and were instructed to adhere to the standard operational protocols developed by LSM before the trial's initiation.

### Data management and confidentiality

Data will be collected in physical records by the study outcome assessors and stored in a two-level locker room in the research facility. All the data will be entered into a database in the REDCap platform. Outcome variables will be entered in duplicate to ensure data quality. To maintain confidentiality, study participants will be identified using an identification number (ID), and only the authors of the study will have access to identification information (i.e., names).

### Missing appointments, early withdrawal, and loss of follow-up of participants

In order to reduce the number of missed appointments, a standardized message will be sent 1 day prior to the scheduled appointment to remember and confirm attendance. If a participant misses an appointment, they will be contacted by phone call to reschedule, with a maximum tolerance period of 14 days. To minimize dropouts and missing data, all eligible participants will be informed of the importance of attending follow-up visits during the baseline visit and in every follow-up phone call. If a participant requests early withdrawal from the study, they will be asked to attend to final evaluation to collect endpoint measurements.

Before considering a participant as unresponsive and proceeding to exclude them from the study due to a lack of contact we will conduct 9 phone call attempts (three phone calls at three different moments), in addition to attempts to contact them through WhatsApp messages. Also, three Smart Speaker EchoDot 3rd Gen (Amazon Echo®) devices will be randomly awarded as incentives among participants who successfully complete the study. This strategy aims to encourage participants to provide complete data, thus improving the overall data quality of the study.

### Sample size

Sample size calculations were performed using the Statistics and Sample Size Pro software [14]. With an alpha level of 0.05, a power of 90%, and an effect size of 0.68 (Cohen’s *d*), it was estimated that 94 subjects (47 per group) would be required to assess mean differences in the primary outcome. Assuming a 20% dropout rate, we plan to include 112 subjects (56 per group) in the study. The effect size of 0.68 was based on the randomized controlled trial published by Fulmer R et al. [15], which evaluated the efficacy of using psychological artificial intelligence software to reduce self-identified symptoms of depression and anxiety. In the referenced trial, the main outcome was assessed using the Patient Health Questionnaire-9 (PHQ-9), which was shown to be equivalent to the Self-Reporting Questionnaire (SRQ-20) used in our trial [16].

## Statistical analysis plan

This manuscript serves as the formal statistical analysis plan (version 1.0) for the trial. The detailed statistical analysis plan (SAP) was elaborated following the guidance published by Gamble et al. (2017) [17], and the SAP checklist is available (Supplementary Table S9).

### Descriptive statistics and baseline data

A consolidated standards of reporting trials (CONSORT) style flow diagram will present the number of patients screened and all reasons for exclusions prior to randomization [18]. Demographic and socioeconomic information (including age, sex, race, socioeconomic status, and educational level), baseline clinical characteristics (such as body mass index [BMI], glycated hemoglobin, systolic and diastolic blood pressure, insulin usage, smoking status, alcohol consumption, comorbidities, performance on up and go, gait speed, and sit and stand tests, and others), as well as other variables, will be described by arm and overall. Categorical variables will be described as frequency (percentage). Continuous variables will be described as mean ± standard deviation (SD) or median and interquartile range. Hypothesis testing for differences in baseline characteristics between the intervention and control arms will not be performed, in accordance with the CONSORT guideline.

### Statistical methods for primary and secondary outcomes

All analysis will be performed after the end of the trial and no interim analysis will be performed. The analysis of all study outcomes will be carried out using analysis of covariance (ANCOVA), as all the outcomes are quantitative. This approach will allow a comparison of means between groups, while also considering potential variations in covariates. As we will compare the means at the end of the study, rather than deltas (differences from baseline), the model will include baseline data of the outcome variable as a covariate. This adjustment allows us to account for the initial variation in scores among the groups before assessing any potential intervention effects.

All analyses will be performed using the intention-to-treat approach, including all subjects who were randomized. Per-protocol analysis will only be performed as a sensitivity analysis, as described in the following section. Normality tests for outcome data will not be performed. Instead, we will conduct visual histogram and QQ plot analyses. If distributional assumptions are in doubt, we will perform a logarithmic transformation of the data before analysis.

### Imputation of missing data

The imputation of missing data will be handled following the European Medicines Agency Guideline on Missing Data in Confirmatory Clinical Trials [19]. The overall rate of missing data at week 12 is expected to be no more than 20% and the frequency and reasons for missing data are expected to be missing at random (MAR). However, the possibility of missing not at random (MNAR) cannot be excluded, as we believe that three factors may influence the missing data:

1. Participants less familiar with technology may have a higher tendency to drop out and might experience reduced benefits due to a higher likelihood of non-adherence to the intervention.
2. Participants from the usual care group may have a higher tendency to discontinue participation due to not receiving the device as they expected.
3. Older participants, who are less likely to experience intervention-related benefits, may be more likely to miss the final evaluation due to potential mobility challenges and a greater reliance on caregiver assistance to attend the consultation.

Therefore, to explore the potential impact of missing data in different scenarios, promoting a better understanding of how different methods of handling missing data impact our results considering the challenge of handling missing data within the possibility of MNAR assumption, three distinct imputation strategies will be employed to assess the impact of missing data on our results:

1. *Multiple imputation with baseline data correction:* we will perform multiple imputation of missing data correcting for the baseline data. This correction aims to mitigate potential bias that could be introduced by missing data imputation using multiple imputation under a possible MNAR assumption.
2. *Last Observation Carried Forward (LOCF):* given our anticipation and belief that both control and intervention subjects are likely to exhibit improvement regarding the baseline parameters, we will adopt the LOCF technique as a sensitivity analysis. By carrying forward the baseline observation, our analysis will be conducted within a more conservative scenario that tends toward the null hypothesis, reducing the probability of type 1 error related to missing data imputation.
3. *Exclusion of participants with missing data:* furthermore, we will conduct a sensitivity analysis considering only the trial completers. As this analysis has the potential to introduce selection bias, the results will be treated as hypothesis generation as subjects with missing data may differ in certain aspects from those without missing data.

### Adjustment for covariates

For adjustment, age, sex, mini-mental state examination score, education, income, and baseline data of the outcome variable will be included in the ANCOVA model as covariates. Additionally, other variables that were found to be imbalanced between groups at baseline will also be included into the analysis.

### Sensitivity and subgroup analyses

Sensitivity analysis will be conducted to assess the impact of missing data as described above. Also, pre-specified subgroup analysis will be performed: age (< 80 vs. ≥ 80 years old), education level (graduate vs. undergraduate), sex (male vs. female), insulin usage (yes vs. no), history of depression (yes vs. no), and cognitive impairment (yes vs. no, accordingly to mini-mental state examination score adjusted for education level). Due to the increased risk of type I and type II errors and reduced power, only subgroup analysis for primary outcome will be performed.

### Adjustment for multiple testing

No adjustment for multiple testing will be performed. Therefore, all outcomes rather than primary outcomes will be considered hypothesis generation and *p*-value will not be reported.

### Confidence intervals, *p*-value, and reporting conventions

The statistical significance level for the primary outcome will be at the 0.05 level (*α* = 0.05). Ninety-five percent confidence intervals for all study outcomes, as well as for all subgroup and sensitivity analyses, will be reported. *P*-value values ≥ 0.001 will be reported to 3 decimal places; values < 0.001 will be reported as “ < 0.001”. Mean and SD values will be reported using one decimal place greater than the original data. Median and quantiles will use the same number of decimal places as the original data.

### Adherence to the intervention

As a pragmatic trial aimed to evaluate the intervention effectiveness in a setting closer to a real-world scenario, the evaluation of outcomes will not consider the patient’s adherence to the proposed intervention, maintaining the integrity of the intention-to-treat analysis.

### Statistical software

The statistical software RStudio (version 4.3.1 or above) and IBM® SPSS Statistics (version 27.0.1 or above) will be used for all the analyses.

## Monitoring and additional information

### Composition of the coordinating center and trial steering committee

The Diabetes and Endocrinology Research Group at the Pontifical Catholic University of Rio Grande do Sul will serve as the coordinating center and trial steering committee for this study.

### Trial sponsor

The senior investigator of the trial (GHT) serves as the Sponsor-investigator for this study. They are responsible for ensuring that research ethics principles are followed, supervision, study design, arranging the financing, data collection, statistical analysis, report writing, and the decision to submit for publication.

### Frequency and plans for auditing trial conduct

No formal audits are planned for this study. Instead, the sponsor-investigator will ensure that the study is implemented in accordance with the protocol and will promptly inform the Research Ethics Committee of Pontifícia Universidade Católica do Rio Grande do Sul of any potential risks that arise during the study. The trial steering committee will convene at least once a month to assess the progress of the study. Due to the short duration of the trial, the small sample size, and the minimal risk to participants, a separate data monitoring committee was not proposed.

### Communication of important protocol modifications

If necessary, protocol modifications will be reported to the Research Ethics Committee of Pontifícia Universidade Católica do Rio Grande do Sul and made publicly available by updating the ClinicalTrials.gov registry by the Sponsor-Investigator. Additionally, any deviations from this study protocol will be reported and justified in all publications related to the study.

## Discussion

To the best of our knowledge, this is the first study to investigate whether an interactive virtual assistance system is effective in improving mental health, glycemic control, and the quality of life in elderly individuals with type 2 diabetes. We believe that it could be a very useful tool, as some disabilities, like physical ones, will not be a criterion of disparity since the system can be used remotely through voice interactions. Thus, findings from our study can potentially open up possibilities for incorporating smart speakers as a valuable tool to enhance the multidisciplinary approach to elderly individuals with diabetes, with significant implications for various stakeholders across the healthcare spectrum. It may assist healthcare providers in facilitating regular communication, medication reminders, and health education for elderly patients, thereby enhancing support for this population. They also have the potential to have a positive impact on reducing the burden on healthcare systems by promoting self-care management and coping mechanisms to deal with geriatric syndromes. Additionally, by embracing technology elderly patients can feel more connected and included in society, mitigating ageism, and promoting a sense of belonging.

## Trial status

This is a protocol (version 2.0, June 2023) for an ongoing trial that started recruitment on June 20, 2023, and is expected to end by May 30, 2024.

### Supplementary Information

**Additional file1:** **Table S1.** SPIRIT Checklist. **Table S2.** Description of the 28 daily messages repeated every 4 weeks translated to English. **Table S3.** Description of the 28 daily messages repeated every 4 weeks in Brazilian Portuguese. **Table S4.** Description of the 7 sets of 14 messages repeated every week translated to English. **Table S5.** Description of the 7 sets of 14 messages repeated every week translated in Brazilian Portuguese. **Table S6.** Description of the content featured in the 12 podcast episodes played weekly by the device. **Table S7.** Logistic and assessments of each visit during the study period. **Table S8.** Logistic of phone calls during the study period. **Table S9.** SAP Checklist.

## Data Availability

Additional information (i.e., data set, statistical code, written consent form, and other protocol details) about the study will be available upon request to the corresponding author after the completion of the study.

## Abbreviations

SRQ-20: Self Reporting Questionnaire 20
ANCOVA: Analysis of covariance
SPIRIT: Standard Protocol Items: Recommendations for Interventional Trials
HSL: Hospital São Lucas
SF-36: 36-Item Short Form Health Survey Questionnaire
SCI-R: Self-Care Inventory Revised Questionnaire
PSS: Perceived Stress Scale
HDL-c: High-density lipoprotein cholesterol
LDL-c: Low-density lipoprotein cholesterol
ID: Identification
PHQ-9: Patient Health Questionnaire-9
SAP: Statistical analysis plan
CONSORT: Consolidated Standards of Reporting Trials
BMI: Body mass index
SD: Standard deviation
MAR: Missing at random
MNAR: Missing not at random
LOCF: Last Observation Carried Forward
CNPq: Brazilian National Council for Scientific and Technological Development
CAPES: Coordination for the Improvement of Higher Education Personnel
