# Supplementary material for: Interactive virtual assistance for mental health promotion and self-care management in elderly with type 2 diabetes (IVAM-ED): study protocol and statistical analysis plan for a randomized controlled trial
Source: Trials. 2024 Mar 21;25:205. doi: 10.1186/s13063-024-08055-3 (PMC10956275; doi:10.1186/s13063-024-08055-3)
Supplement: Supplementary file 1 — Additional file1: Table S1. SPIRIT Checklist. Table S2. Description of the 28 daily messages repeated every 4 weeks translated to English. Table S3. Description of the 28 daily messages repeated every 4 weeks in Brazilian Portuguese. Table S4. Description of the 7 sets of 14 messages repeated every week translated to English. Table S5. Description of the 7 sets of 14 messages repeated every week translated in Brazilian Portuguese. Table S6. Description of the content featured in the 12 podcast episodes played weekly by the device. Table S7. Logistic and assessments of each visit during the study period. Table S8. Logistic of phone calls during the study period. Table S9. SAP Checklist. [file 13063_2024_8055_MOESM1_ESM.docx]

**SUPPLEMENTARY MATERIAL**

***Interactive Virtual Assistance for Mental Health Promotion and Self-Care Management in Elderly with Type 2 Diabetes (IVAM-ED): study protocol and statistical analysis plan for a randomized controlled trial***

**SUMMARY**

[Table S1. SPIRIT Checklist 1](#_Toc153490744)

[Table S2. Description of the 28 daily messages repeated every 4 weeks translated to English 1](#_Toc153490745)

[Table S5. Description of the 28 daily messages repeated every 4 weeks in Brazilian Portuguese 3](#_Toc153490746)

[Table S4. Description of the 7 sets of 14 messages repeated every week translated to English 5](#_Toc153490747)

[Table S5. Description of the 7 sets of 14 messages repeated every week translated in Brazilian Portuguese 6](#_Toc153490748)

[Table S6. Description of the content featured in the 12 podcast episodes played weekly by the device 7](#_Toc153490749)

[Table S7. Logistic and assessments of each visit during the study period 8](#_Toc153490750)

[Table S8. Logistic of phone calls during the study period 9](#_Toc153490751)

[Table S9. SAP Checklist 1](#_Toc153490752)0

# Table S1. SPIRIT Checklist

| Section/item | Item No | Description | Location where item is reported |
| --- | --- | --- | --- |
| **Administrative information** | | | |
| Title | 1 | Descriptive title identifying the study design, population, interventions, and, if applicable, trial acronym | p. 01 |
| Trial registration | 2a | Trial identifier and registry name. If not yet registered, name of intended registry | p. 02 |
|  | 2b | All items from the World Health Organization Trial Registration Data Set | p. 02 |
| Protocol version | 3 | Date and version identifier | p. 02 |
| Funding | 4 | Sources and types of financial, material, and other support | p. 16 |
| Roles and responsibilities | 5a | Names, affiliations, and roles of protocol contributors | p. 01, p. 16 |
|  | 5b | Name and contact information for the trial sponsor | p. 16 |
|  | 5c | Role of study sponsor and funders, if any, in study design; collection, management, analysis, and interpretation of data; writing of the report; and the decision to submit the report for publication, including whether they will have ultimate authority over any of these activities | p. 16 |
|  | 5d | Composition, roles, and responsibilities of the coordinating centre, steering committee, endpoint adjudication committee, data management team, and other individuals or groups overseeing the trial, if applicable (see Item 21a for data monitoring committee) | p. 19 |
| **Introduction** | | | |
| Background and rationale | 6a | Description of research question and justification for undertaking the trial, including summary of relevant studies (published and unpublished) examining benefits and harms for each intervention | p. 03 |
|  | 6b | Explanation for choice of comparators | p. 03 |
| Objectives | 7 | Specific objectives or hypotheses | p. 04 |
| Trial design | 8 | Description of trial design including type of trial (eg, parallel group, crossover, factorial, single group), allocation ratio, and framework (eg, superiority, equivalence, noninferiority, exploratory) | p. 04 |
| Methods: Participants, interventions, and outcomes | | | |
| Study setting | 9 | Description of study settings (eg, community clinic, academic hospital) and list of countries where data will be collected. Reference to where list of study sites can be obtained | p. 04 |
| Eligibility criteria | 10 | Inclusion and exclusion criteria for participants. If applicable, eligibility criteria for study centres and individuals who will perform the interventions (eg, surgeons, psychotherapists) | p. 05 |
| Interventions | 11a | Interventions for each group with sufficient detail to allow replication, including how and when they will be administered | p. 06 |
|  | 11b | Criteria for discontinuing or modifying allocated interventions for a given trial participant (eg, drug dose change in response to harms, participant request, or improving/worsening disease) | p. 09, p. 13 |
|  | 11c | Strategies to improve adherence to intervention protocols, and any procedures for monitoring adherence (eg, drug tablet return, laboratory tests) | p. 09, p. 13 |
|  | 11d | Relevant concomitant care and interventions that are permitted or prohibited during the trial | p. 07 |
| Outcomes | 12 | Primary, secondary, and other outcomes, including the specific measurement variable (eg, systolic blood pressure), analysis metric (eg, change from baseline, final value, time to event), method of aggregation (eg, median, proportion), and time point for each outcome. Explanation of the clinical relevance of chosen efficacy and harm outcomes is strongly recommended | p. 08-09, p. 11 |
| Participant timeline | 13 | Time schedule of enrolment, interventions (including any run-ins and washouts), assessments, and visits for participants. A schematic diagram is highly recommended (see Figure) | p. 08 |
| Sample size | 14 | Estimated number of participants needed to achieve study objectives and how it was determined, including clinical and statistical assumptions supporting any sample size calculations | p. 09 |
| Recruitment | 15 | Strategies for achieving adequate participant enrolment to reach target sample size | p. 04 |
| **Methods: Assignment of interventions (for controlled trials)** | | | |
| Allocation: | | | |
| Sequence generation | 16a | Method of generating the allocation sequence (eg, computer-generated random numbers), and list of any factors for stratification. To reduce predictability of a random sequence, details of any planned restriction (eg, blocking) should be provided in a separate document that is unavailable to those who enrol participants or assign interventions | p. 05 |
| Allocation concealment mechanism | 16b | Mechanism of implementing the allocation sequence (eg, central telephone; sequentially numbered, opaque, sealed envelopes), describing any steps to conceal the sequence until interventions are assigned | p. 05 |
| Implementation | 16c | Who will generate the allocation sequence, who will enrol participants, and who will assign participants to interventions | p. 05 |
| Blinding (masking) | 17a | Who will be blinded after assignment to interventions (eg, trial participants, care providers, outcome assessors, data analysts), and how | p. 05 |
|  | 17b | If blinded, circumstances under which unblinding is permissible, and procedure for revealing a participant’s allocated intervention during the trial | p. 05 |
| **Methods: Data collection, management, and analysis** | | | |
| Data collection methods | 18a | Plans for assessment and collection of outcome, baseline, and other trial data, including any related processes to promote data quality (eg, duplicate measurements, training of assessors) and a description of study instruments (eg, questionnaires, laboratory tests) along with their reliability and validity, if known. Reference to where data collection forms can be found, if not in the protocol | p. 08-09, p. 13 |
|  | 18b | Plans to promote participant retention and complete follow-up, including list of any outcome data to be collected for participants who discontinue or deviate from intervention protocols | p. 09 |
| Data management | 19 | Plans for data entry, coding, security, and storage, including any related processes to promote data quality (eg, double data entry; range checks for data values). Reference to where details of data management procedures can be found, if not in the protocol | p. 09 |
| Statistical methods | 20a | Statistical methods for analysing primary and secondary outcomes. Reference to where other details of the statistical analysis plan can be found, if not in the protocol | p. 10-11 |
|  | 20b | Methods for any additional analyses (eg, subgroup and adjusted analyses) | p. 11 |
|  | 20c | Definition of analysis population relating to protocol non-adherence (eg, as randomised analysis), and any statistical methods to handle missing data (eg, multiple imputation) | p. 10 |
| **Methods: Monitoring** | | | |
| Data monitoring | 21a | Composition of data monitoring committee (DMC); summary of its role and reporting structure; statement of whether it is independent from the sponsor and competing interests; and reference to where further details about its charter can be found, if not in the protocol. Alternatively, an explanation of why a DMC is not needed | p. 19 |
|  | 21b | Description of any interim analyses and stopping guidelines, including who will have access to these interim results and make the final decision to terminate the trial | p. 11 |
| Harms | 22 | Plans for collecting, assessing, reporting, and managing solicited and spontaneously reported adverse events and other unintended effects of trial interventions or trial conduct | p. 08 |
| Auditing | 23 | Frequency and procedures for auditing trial conduct, if any, and whether the process will be independent from investigators and the sponsor | p. 19 |
| Ethics and dissemination | | | |
| Research ethics approval | 24 | Plans for seeking research ethics committee/institutional review board (REC/IRB) approval | p. 15 |
| Protocol amendments | 25 | Plans for communicating important protocol modifications (eg, changes to eligibility criteria, outcomes, analyses) to relevant parties (eg, investigators, REC/IRBs, trial participants, trial registries, journals, regulators) | p. 19 |
| Consent or assent | 26a | Who will obtain informed consent or assent from potential trial participants or authorised surrogates, and how (see Item 32) | p. 15 |
|  | 26b | Additional consent provisions for collection and use of participant data and biological specimens in ancillary studies, if applicable | N/A - No biological specimens were collected as part of the trial |
| Confidentiality | 27 | How personal information about potential and enrolled participants will be collected, shared, and maintained in order to protect confidentiality before, during, and after the trial | p. 09 |
| Declaration of interests | 28 | Financial and other competing interests for principal investigators for the overall trial and each study site | p. 15 |
| Access to data | 29 | Statement of who will have access to the final trial dataset, and disclosure of contractual agreements that limit such access for investigators | p. 09 |
| Ancillary and post-trial care | 30 | Provisions, if any, for ancillary and post-trial care, and for compensation to those who suffer harm from trial participation | N/A – No provisions for post-trial care or compensation are anticipated |
| Dissemination policy | 31a | Plans for investigators and sponsor to communicate trial results to participants, healthcare professionals, the public, and other relevant groups (eg, via publication, reporting in results databases, or other data sharing arrangements), including any publication restrictions | p. 15 |
|  | 31b | Authorship eligibility guidelines and any intended use of professional writers | p. 15 |
|  | 31c | Plans, if any, for granting public access to the full protocol, participant-level dataset, and statistical code | p. 15 |
| **Appendices** | | | |
| Informed consent materials | 32 | Model consent form and other related documentation given to participants and authorised surrogates | p. 15 |
| Biological specimens | 33 | Plans for collection, laboratory evaluation, and storage of biological specimens for genetic or molecular analysis in the current trial and for future use in ancillary studies, if applicable | N/A - No biological specimens were collected as part of the trial |

# Table S2. Description of the 28 daily messages repeated every 4 weeks translated to English

| **No** | **Content** |
| --- | --- |
| 1 | Take some time for yourself today. Perhaps go read a book, magazine, or newspaper. |
| 2 | Do an act of kindness for yourself today; go and take a relaxing bath or have a hot cup of tea now. |
| 3 | It's important to have moments of relaxation and leisure activities in your daily routine. Try to find some time in your day to do something you enjoy. |
| 4 | Maintaining contact with friends and family, including through regular phone calls, is important. How about calling a loved one now? |
| 5 | How about challenging your mind by solving memory problems? I can help you with that through a quiz. If you're interested, just say, "Alexa, open My Quiz." |
| 6 | Enjoy the early morning or late afternoon sun to get some vitamin D. |
| 7 | How about taking a break now and revisiting old photos of special moments? Reliving positive memories is important and can be uplifting. |
| 8 | Consider watching a movie or TV show that brings you joy or that you like. |
| 9 | Why not try a healthy cooking recipe today? |
| 10 | When eating, opt for whole foods such as whole grain bread and cereals instead of refined carbohydrates. |
| 11 | How about challenging yourself to increase your consumption of vegetables like lettuce, kale, and spinach this week? These are fiber-rich and nutrient-rich foods that can help with diabetes management. |
| 12 | It's important to limit the consumption of processed foods like snacks and frozen meals. |
| 13 | You can reduce the salt in your food by using natural seasonings like herbs, spices, and black pepper. How about trying to make a dish with less salt today? |
| 14 | You can replace sugary desserts with healthier options like fresh fruits or plain yogurt. |
| 15 | Eat small portions of food at each meal and avoid eating large quantities at once. |
| 16 | Have balanced meals, including a source of protein or meat, vegetables, few carbohydrates, and low fats. |
| 17 | Read food labels and avoid those that contain a lot of sugars and unhealthy ingredients. |
| 18 | Whenever possible, cook your meals at home to have control over the ingredients and preparation. |
| 19 | Whenever you check your blood glucose level or have a hypoglycemia episode, write it down in a notebook with the date and value. |
| 20 | If you have a hypoglycemia episode, remember that just a tablespoon of sugar is enough to reverse it. Be careful, as excess sugar can also be a problem. |
| 21 | Have an emergency plan for hypoglycemia, and remember to take something to eat when you leave home. |
| 22 | Maintain an adequate stock of diabetes-related medications and supplies, such as glucose test strips, medications, and insulin needles, to avoid interruptions in your treatment. |
| 23 | How about learning relaxation techniques like deep breathing and meditation to cope with stress? I can help you with that; just ask, "Alexa, open the meditation skill." |
| 24 | Sometimes, it's normal to feel that having diabetes is a burden, and these emotions are part of the process of learning to cope with the disease. Pay attention to your mental and emotional health. |
| 25 | Diabetes is a manageable condition; with proper treatment, you can keep your blood glucose levels in check and lead a good and normal life. |
| 26 | Seek support from family, friends, and support groups to share experiences, tips, and challenges related to diabetes treatment. Having people who understand and support you can make a big difference. |
| 27 | The goal of diabetes treatment is to improve your quality of life and overall well-being. Focus on taking care of yourself and making healthy choices that bring long-term benefits to your life. |
| 28 | Even with a diabetes diagnosis, you don't have to give up all the things you love or the foods you enjoy. |

# Table S5. Description of the 28 daily messages repeated every 4 weeks in Brazilian Portuguese

| **No** | **Content** |
| --- | --- |
| 1 | Separe um tempo do seu dia para você. Quem sabe vá ler um livro, revista ou jornal |
| 2 | Faça um ato de gentileza consigo mesmo hoje, vá e tome um banho relaxante ou tome uma xícara de chá quente agora |
| 3 | É importante ter momentos de relaxamento e atividades de lazer em sua rotina diária. Procure encontrar um tempo no seu dia para fazer algo que você gosta |
| 4 | É importante manter contato com amigos e familiares, inclusive por meio de chamadas telefônicas regulares. O que acha de ligar para alguém querido agora? |
| 5 | Que tal estimular a sua mente resolvendo problemas de memória? Eu posso lhe ajudar com isso com um jogo de perguntas. Caso tenha interesse basta dizer: “Alexa abrir Meu Quiz” |
| 6 | Aproveite o sol da manhã cedo ou no final da tarde para obter um pouco de vitamina D |
| 7 | Que tal fazer uma pausa agora e rever fotos antigas de momentos especiais? Reviver memórias positivas é importante e pode fazer bem |
| 8 | Que tal assistir algum filme ou novela que lhe traga alegria ou que você goste? |
| 9 | Que tal experimentar uma receita de culinária saudável hoje? |
| 10 | Ao se alimentar, opte por alimentos integrais, como pães e cereais, ao invés de alimentos com carboidratos refinados |
| 11 | Que tal se desafiar e tentar aumentar o consumo de vegetais, como alface, couve e espinafre essa semana? São alimentos ricos em fibras e nutrientes que podem ajudar com o controle da diabetes |
| 12 | É importante limitar o consumo de alimentos processados como salgadinhos e alimentos congelados |
| 13 | Você pode reduzir o consumo de sal utilizado nos alimentos substituindo-o por outros temperos naturais como ervas, especiarias e pimenta do reino. Que tal tentar fazer um prato com menos sal hoje? |
| 14 | Você pode substituir sobremesas açucaradas por opções mais saudáveis como frutas frescas ou iogurte natural |
| 15 | Você pode substituir sobremesas açucaradas por opções mais saudáveis como frutas frescas ou iogurte natural |
| 16 | Faça refeições equilibradas, incluindo uma fonte de proteína ou carne, vegetais, poucos carboidratos e poucas gorduras |
| 17 | Leia os rótulos dos alimentos e evite aqueles que contêm muito açúcares e ingredientes não saudáveis |
| 18 | Faça refeições caseiras sempre que possível para ter controle dos ingredientes e do preparo |
| 19 | Sempre que verificar o nível de glicose no sangue ou tiver um episódio de hipoglicemia, anote em um caderninho com a data e o valor |
| 20 | Caso tenha um episódio de hipoglicemia lembre-se que basta uma colher de sopa de açúcar para reverter. Cuidado pois o excesso de açúcar também pode ser um problema |
| 21 | Tenha um plano de emergência em caso de hipoglicemia, lembre-se de levar algo para comer quando sair de casa |
| 22 | Mantenha um estoque adequado de medicamentos e suprimentos relacionados ao diabetes, como fitas de HGT, medicações e agulhas de insulina para evitar interrupções no tratamento |
| 23 | Que tal aprender técnicas de relaxamento, como respiração profunda e meditação, para lidar com o estresse? Posso te ajudar com isso, basta solicitar: Alexa, abra a skill de meditação |
| 24 | Às vezes é normal sentir que ter diabetes é um peso e essas emoções fazem parte do processo de aprender a lidar com a doença. Dê atenção à sua saúde mental e emocional |
| 25 | O diabetes é uma condição controlável; com o tratamento adequado, você pode manter os níveis de glicose no sangue sob controle e levar uma vida boa e normal |
| 26 | Busque apoio de familiares, amigos e grupos de apoio para compartilhar experiências, dicas e desafios relacionados com o tratamento de diabetes. Ter pessoas que entendam e apoiem você pode fazer toda a diferença |
| 27 | O objetivo do tratamento do diabetes é melhorar sua qualidade de vida e bem-estar geral. Concentre-se em cuidar de si mesmo e fazer escolhas saudáveis que tragam benefícios a longo prazo para sua vida |
| 28 | Mesmo com o diagnóstico de diabetes você não precisa abrir mão de todas as coisas que ama e de comer as coisas que gosta |

# Table S4. Description of the 7 sets of 14 messages repeated every week translated to English

| **No** | **Content** |
| --- | --- |
| 1 | If possible, engage in a stretching session. |
|  | Engaging in moderate-intensity physical activities for 30 minutes a day is enough to stop being sedentary. These 30 minutes can be divided into 10-minute periods. Always strive to stay active. |
| 2 | The most common symptoms of hypoglycemia are: tremors, dizziness, excessive sweating, fainting. Always remember to pay attention to the presence of these symptoms. |
|  | Make sure to use the correct insulin doses and always administer them at the time recommended by your medical team. |
| 3 | Remember to take care of your feet, be careful not to injure them, and always check for the presence of lesions or wounds. Take care of your footwear and always make sure they are suitable for use. |
|  | Maintain regular medical check-ups, perform your blood glucose tests as directed by your medical team, and make sure to use prescribed medications correctly. |
| 4 | In addition, reducing the use of electronic devices with screens a few hours before bedtime is important. All of these actions will help you sleep better. |
|  | Avoid drinking coffee and mate (chimarrão) after 5 PM; these beverages contain caffeine and can disrupt your sleep. |
| 5 | Remember to drink water regularly. |
|  | Avoid naps after 5 PM, as it may make it easier to sleep at night. |
| 6 | Feeling lonely? How about sending a message or calling a friend or family member now? |
|  | Remember to set achievable daily goals and celebrate small achievements. |
| 7 | Shall we meditate now? If you're interested, just ask for the meditation skill. |
|  | Listening to music you enjoy can boost your spirits and well-being. I can play any music for you, just ask: "Alexa, play music." |

# Table S5. Description of the 7 sets of 14 messages repeated every week translated in Brazilian Portuguese

| **No** | **Content** |
| --- | --- |
| 1 | Se possível, realize uma sessão de alongamento |
|  | Praticar atividades físicas de intensidade moderada por 30 minutos por dia é o suficiente para deixar de ser sedentário. Esses 30 minutos podem ser divididos em períodos de 10 minutos. Procure sempre se manter ativo. |
| 2 | Os sintomas mais comuns de hipoglicemia são: tremores, tonturas, suor intenso, desmaio. Lembre-se de sempre cuidar a presença desses sintomas. |
|  | Procure utilizar as doses corretas de insulina e sempre aplicar no horário orientado pela sua equipe médica |
| 3 | Lembre-se de tomar cuidado com seus pés, cuide para não se machucar e sempre verifique a presença de lesões ou feridas. Cuide seus calçados e sempre confira se estão adequados para uso. |
|  | Mantenha acompanhamento médico regular, faça seu HGT conforme orientação da sua equipe médica e procure usar corretamente as medicações prescritas |
| 4 | Evite beber café e chimarrão após as 17 horas; essas bebidas possuem cafeína e podem atrapalhar o seu sono |
|  | Além disso, diminuir o uso de dispositivos eletrônicos com telas algumas horas antes de dormir é importante. Todas essas atitudes irão ajudar você a dormir melhor |
| 5 | Lembre-se de beber água regularmente |
|  | Evitar cochilos após as 17 horas pode fazer com que seja mais fácil dormir à noite |
| 6 | Está se sentindo sozinho? Que tal mandar uma mensagem ou telefonar para algum amigo ou familiar agora? |
|  | Lembre-se de criar metas diárias alcançáveis e de comemorar pequenas conquistas |
| 7 | Vamos meditar agora? Caso tenha interesse, basta pedir pela skill de meditação |
|  | Escutar uma música que você goste pode estimular o ânimo e o bem-estar. Posso tocar qualquer música para você, basta pedir: “Alexa, toque música" |

# Table S6. Description of the content featured in the 12 podcast episodes played weekly by the device

| **Subject** | **Episode** | **Description** |
| --- | --- | --- |
| Physical Exercise | 1 | Benefits of physical exercise: glycemic control, weight loss, pain reduction, improved mental health and overall quality of life improvement |
|  | 2 | Weekly goals for physical exercise |
|  | 3 | Appropriate exercise modalities for seniors and strategies to achieve physical exercise goals |
| Diabetes Selfcare | 1 | Foot care and fall prevention. |
|  | 2 | Glycemic control and hypoglycemia management |
|  | 3 | Strategies to improve diabetes-related self-care |
| Mental Health | 1 | How sleeping impacts on mental health, quality of life, sleep hygiene and habits that improve its quality |
|  | 2 | Anxiety and depression management and importance of maintaining a social circle |
|  | 3 | Reflections on aging and the process of the finiteness of life |
| Healthy Eating Habits | 1 | Glycemic index and benefits of including fiber and protein in the diet |
|  | 2 | Strategies to achieve a balanced diet and make better food choices |
|  | 3 | How to organize a shopping list and identify processed foods to be avoided |

# Table S7. Logistic and assessments of each visit during the study period

| **Visit** | **Baseline Evaluation**  **(week -1)** | **Installation of the device***  **(week 0)** | **Final Evaluation**  **(week 12)** |
| --- | --- | --- | --- |
| Predetermined duration of visit (minutes) | 120 | 15 | 60 |
| Protocol assessment time windows (days) | NA | ±7 | ±7 |
| Identification data | x |  |  |
| Clinical interview and baseline evaluation | x |  |  |
| Clinical evaluation | x |  | X |
| Laboratory evaluation | x |  | X |
| Medication use | x |  | X |
| Installation of device |  | x |  |

*This applies only to participants from the intervention group. Participants in the control group will receive an additional phone call in week 0 to ensure equivalency. NA refers to not applied.

# Table S8. Logistic of phone calls during the study period

| **Phone call** | **Week of study** |
| --- | --- |
| Phone call 0* | Week 0 |
| Phone call 1 | Week 2 |
| Phone call 2 | Week 4 |
| Phone call 3 | Week 6 |
| Phone call 4 | Week 8 |
| Phone call 5 | Week 10 |

*Only for participants from the control group. Equivalent to installation of the device.

# Table S9. SAP Checklist

| Section/Item | Index | Description | Reported on page # |
| --- | --- | --- | --- |
| **Section 1: Administrative information** | | | |
| Trial and Trial registration | 1a | Descriptive title that matches the protocol, with SAP either as a forerunner or subtitle,  and trial acronym (if applicable) | p. 01 |
|  | 1b | Trial registration number | p. 02 |
| SAP Version | 2 | SAP version number with dates | p. 10 |
| Protocol Version | 3 | Reference to version of protocol being used | NA |
| SAP revisions | 4a | SAP revision history | p. 10 |
|  | 4b | Justification for each SAP revision | NA |
|  | 4c | Timing of SAP revisions in relation to interim analyses, etc. | NA |
| Roles and responsibility | 5 | Names, affiliations, and roles of SAP contributors | p. 01, p. 16 |
| Signatures of: | 6a | Person writing the SAP | p. 16 |
|  | 6b | Senior statistician responsible | p. 16 |
|  | 6c | Chief investigator/clinical lead | p. 16 |
| **Section 2: Introduction** | | | |
| Background and rationale | 7 | Synopsis of trial background and rationale including a brief description of research question  and brief justification for undertaking the trial | p. 03 |
| Objectives | 8 | Description of specific objectives or hypotheses | p. 04 |
| **Section 3: Study Methods** | | | |
| Trial design | 9 | Brief description of trial design including type of trial (e.g., parallel group, multi-arm, crossover, factorial) and allocation ratio and may include brief description of interventions | p. 04 |
| Randomization | 10 | Randomization details, e.g., whether any minimization or stratification occurred (including stratifying factors used or the location of that information if it is not held within the SAP) | p. 05 |
| Sample size | 11 | Full sample size calculation or reference to sample size calculation in protocol  (instead of replication in SAP) | p. 10 |
| Framework | 12 | Superiority, equivalence, or noninferiority hypothesis testing framework, including which comparisons will be presented on this basis | p. 04 |
| Statistical interim analysis and stopping guidance | 13a | Information on interim analyses specifying what interim analyses will be carried out  and listing of time points | p. 11 |
|  | 13b | Any planned adjustment of the significance level due to interim analysis | NA |
|  | 13c | Details of guidelines for stopping the trial early | NA |
| Timing of final analysis | 14 | Timing of final analysis, e.g., all outcomes analysed collectively or timing stratified by planned length of follow-up | p. 11 |
| Timing of outcome assessments | 15 | Time points at which the outcomes are measured including visit “windows” | p. 08, p. 11 |
| **Section 4: Statistical Principals** | | | |
| Confidence intervals and *P* values | 16 | Level of statistical significance | p. 13 |
|  | 17 | Description and rationale for any adjustment for multiplicity and, if so, detailing how the type 1 error is to be controlled | p. 13 |
|  | 18 | Confidence intervals to be reported | p. 13 |
| Adherence and Protocol deviations | 19a | Definition of adherence to the intervention and how this is assessed including extent of exposure | p. 13 |
|  | 19b | Description of how adherence to the intervention will be presented | p. 13 |
|  | 19c | Definition of protocol deviations for the trial | p. 13 |
|  | 19d | Description of which protocol deviations will be summarized | p. 13 |
| Analysis populations | 20 | Definition of analysis populations, e.g., intention to treat, per protocol, complete case, safety | p. 11 |
| **Section 5: Trial Population** | | | |
| Screening data | 21 | Reporting of screening data (if collected) to describe representativeness of trial sample | p. 10 |
| Eligibility | 22 | Summary of eligibility criteria | p. 05 |
| Recruitment | 23 | Information to be included in the CONSORT flow diagram | p. 10 |
| Withdrawal/ Follow-up | 24a | Level of withdrawal, e.g., from intervention and/or from follow-up | p. 09 |
|  | 24b | Timing of withdrawal/lost to follow-up data | p. 09 |
|  | 24c | Reasons and details of how withdrawal/lost to follow-up data will be presented | p. 09 |
| Baseline patient characteristics | 25a | List of baseline characteristics to be summarized | p. 10 |
|  | 25b | Details of how baseline characteristics will be descriptively summarized | p. 10 |
| **Section 6: Analysis** | | | |
| Outcome definitions | List and describe each primary and secondary outcome including details of: | |  |
|  | 26a | Specification of outcomes and timings. If applicable include the order of importance of primary  or key secondary end points (e.g., order in which they will be tested) | p. 08, p. 11 |
|  | 26b | Specific measurement and units (e.g., glucose control, hbA1c [mmol/mol or %]) | p. 08 |
|  | 26c | Any calculation or transformation used to derive the outcome (e.g., change from baseline, QoL score,Time to event, logarithm, etc.) | p. 11 |
| Analysis methods | 27a | What analysis method will be used and how the treatment effects will be presented | p. 11 |
|  | 27b | Any adjustment for covariates | p. 12 |
|  | 27c | Methods used for assumptions to be checked for statistical methods | p. 11 |
|  | 27d | Details of alternative methods to be used if distributional assumptions do not hold, e.g., normality, proportional hazards, etc. | p. 11 |
|  | 27e | Any planned sensitivity analyses for each outcome where applicable | p. 13 |
|  | 27f | Any planned subgroup analyses for each outcome including how subgroups are defined | p. 13 |
| Missing data | 28 | Reporting and assumptions/statistical methods to handle missing data (e.g., multiple imputation) | p. 12 |
| Additional analyses | 29 | Details of any additional statistical analyses required, e.g., complier-average causal effect10 analysis | NA |
| Harms | 30 | Sufficient detail on summarizing safety data, e.g., information on severity, expectedness, and causality; details of how adverse events are coded or categorized; how adverse event data will be analysed, i.e., grade 3/4 only, incidence case analysis, intervention emergent analysis | p. 08 |
| Statistical software | 31 | Details of statistical packages to be used to carry out analyses | p. 13 |
| References | 32a | References to be provided for nonstandard statistical methods | p. 15 |
|  | 32b | Reference to Data Management Plan | NA |
|  | 32c | Reference to the Trial Master File and Statistical Master File | NA |
|  | 32d | Reference to other standard operating procedures or documents to be adhered to | NA |
